# The Protective Effect of Vanadium on Cognitive Impairment and the Neuropathology of Alzheimer’s Disease in APPSwe/PS1dE9 Mice

**DOI:** 10.3389/fnmol.2020.00021

**Published:** 2020-03-10

**Authors:** Zhijun He, Shuangxue Han, Huazhang Zhu, Xia Hu, Xiaoqian Li, Chaofan Hou, Chong Wu, Qingguo Xie, Nan Li, Xiubo Du, Jiazuan Ni, Qiong Liu

**Affiliations:** ^1^Shenzhen Key Laboratory of Marine Biotechnology and Ecology, College of Life Sciences and Oceanography, Shenzhen University, Shenzhen, China; ^2^College of Optoelectronics Engineering, Shenzhen University, Shenzhen, China; ^3^College of Life Science, Huazhong University of Science and Technology, Wuhan, China

**Keywords:** Alzheimer’s disease, amyloid beta (Aβ), vanadium, bis(ethylmaltolato) oxidovanadium (IV) (BEOV), peroxisome proliferator-activated receptor gamma (PPARγ), protein tyrosine phosphatase-1B (PTP1B), autophagy

## Abstract

Alzheimer’s disease (AD) is a widely distributed neurodegenerative disease characterized clinically by cognitive deficits and pathologically by formation of amyloid-β (Aβ) plaque and neurofibrillary tangles (NFTs) in the brain. Vanadium is a biological trace element that has a function to mimic insulin for diabetes. Bis(ethylmaltolato) oxidovanadium (IV) (BEOV) has been reported to have a hypoglycemic property, but its effect on AD remains unclear. In this study, BEOV was supplemented at doses of 0.2 and 1.0 mmol/L to the AD model mice APPSwe/PS1dE9 for 3 months. The results showed that BEOV substantially ameliorated glucose metabolic disorder as well as synaptic and behavioral deficits of the AD mice. Further investigation revealed that BEOV significantly reduced Aβ generation by increasing the expression of peroxisome proliferator-activated receptor gamma and insulin-degrading enzyme and by decreasing β-secretase 1 in the hippocampus and cortex of AD mice. BEOV also reduced tau hyperphosphorylation by inhibiting protein tyrosine phosphatase-1B and regulating the pathway of insulin receptor/insulin receptor substrate-1/protein kinase B/glycogen synthase kinase 3 beta. Furthermore, BEOV could enhance autophagolysosomal fusion and restore autophagic flux to increase the clearance of Aβ deposits and phosphorylated tau in the brains of AD mice. Collectively, the present study provides solid data for revealing the function and mechanism of BEOV on AD pathology.

## Introduction

Alzheimer’s disease (AD), an age-related neurodegenerative disease first described by German neurologist Alois Alzheimer in 1907, is the most common type of dementia worldwide (Vinters, 2015). It is estimated that AD afflicts 5.3 million Americans and inflicts a heavy economic burden on their families and societies (Alzheimer’s Association, 2015). In 2015, AD-related cost was over 600 billion dollars worldwide and over 200 billion dollars in the US alone (Alzheimer’s Association, 2016). If this disease remains unchecked, the number of AD patients is expected to exceed 130 million and the related cost is estimated to be over $1 trillion per year (Honig et al., 2018). Thus, it is extremely important to find a novel strategy for AD treatment.

Neuritic plaques, mainly consisting of β-amyloid (Aβ) aggregates, and neurofibrillary tangles (NFTs), consisting of hyperphosphorylated tau proteins, are considered the two major hallmarks of this progressive neuropathology (Glenner and Wong, 1984; Grundke-Iqbal et al., 1986). Toxic Aβ oligomers and hyperphosphorylated tau aggregates lead to neuronal and synaptic loss and eventually AD-related symptoms (Neniskyte et al., 2011; Akihiko, 2013). Despite intensive research in recent years, the underlying mechanisms and the consequences for AD pathogenesis remain incompletely understood. Clinical and epidemiological studies have consistently shown an intimate relationship between the pathological characteristics of diabetes mellitus (DM) and AD (Arvanitakis et al., 2004; Asih et al., 2017). Type 2 diabetes mellitus (T2DM) increases the risk of developing AD (Akter et al., 2011; Vagelatos and Eslick, 2013) and AD patients also exhibit disturbed energy metabolism and insulin resistant in the brain, possibly due to impairments of insulin signaling (Talbot et al., 2012; Chen and Zhong, 2013). Aβ could bind to insulin receptor (IR; Xie et al., 2002) and impair brain insulin signaling, leading to altered activity of downstream proteins like protein kinase B (Akt) and glycogen synthase kinase 3 beta (GSK3β; Pearson-Leary and McNay, 2012; Zhang et al., 2016). Deletion of tau in mouse has been reported to promote brain insulin resistance (Marciniak et al., 2017). Tau pathology is inferred to induce brain insulin resistance, which is instrumental for cognitive and metabolic impairments in AD patients. Based on the link between AD and T2DM, it is reasonable to think that insulin enhancement agents could have the capacity to prevent AD progression and drugs for T2DM treatment such as the peroxisome proliferator-activated receptor γ (PPARγ) agonists are potential candidates for the treatment of AD (Landreth et al., 2008; Nicolakakis and Hamel, 2010).

Vanadium has been considered as an essential micronutrient within many organisms (Schroeder et al., 1963). Vanadium compounds exhibit insulin-like effects involving stimulation of glucose uptake and inhibition of lipolysis through multiple mechanisms (Heyliger et al., 1985; Li et al., 1997; Reul et al., 1999; Ou et al., 2005; Gao et al., 2008). A recent clinical study demonstrated that elevated insulin levels in the central nervous system of nasal improved the cognition or daily function of patients with AD (Craft et al., 2012; Claxton et al., 2015). Vanadium compounds like vanadyl (IV) acetylacetonate could enhance the IR/PI3K/Akt signaling through inhibiting protein tyrosine phosphatases (PTPs) in differentiated rat adipocytes. Activation of the PPARγ-AMP-activated protein kinase (AMPK) signaling pathway was also found in the APPSwe/PS1dE9 model mice treated with vanadium (Huyer et al., 1997; Wu et al., 2013). Vanadium salts such as vanadyl sulfate or sodium metavanadate have been observed to be antidiabetic, but their side effects reduce their beneficial efficacy. In comparison, organic vanadium compounds dramatically improve the uptake of vanadium (Setyawati et al., 1998; Thompson et al., 2002; Crans et al., 2019). Among them, bis(ethylmaltolato) oxidovanadium (IV) (BEOV) is synthesized from a food additive ethyl maltol and oxovanadium (IV) that has very low toxicity and good anti-diabetic function. However, its effect on AD has rarely been reported.

In this study, the effect of BEOV on AD pathology and its underlying mechanism was investigated in the 9-month-old double transgenic AD model mice APPSwe/PS1dE9 and the AD model cell line N2asw. We found that BEOV could mitigate cognitive impairment and promote glucose uptake of the AD model mice. Furthermore, BEOV could reduce the production of Aβ by regulating the activity of PPARγ and insulin-degrading enzyme (IDE). It also inhibited tau hyperphosphorylation by modulating the activity of protein tyrosine phosphatase-1B (PTP1B) and Akt/GSK3β pathway. Meanwhile, BEOV could increase autophagic flux to promote the clearance of Aβ and tau aggregates in the hippocampus and cortex of the APPSwe/PS1dE9 mice.

## Materials and Methods

### Animals and Pharmacological Treatment

The double transgenic model mice of AD (APPSwe/PS1dE9), co-expressing the Swedish mutation of human amyloid precursor protein (APPswe) and presenilin 1 deleted in exon 9 (PS1-dE9; Garcia-Alloza et al., 2006), and the age/sex-matched non-transgenic C57BL/6N mice (wild-type) were purchased from the Guangdong Medical Lab Animal Center (Guangdong, China). The mice were housed in standard cages (4–5 mice per cage) and maintained in a humidity- and temperature-controlled colony room with 12-h light/12-h dark cycle and free access to water and food. All animal experiments were performed in accordance with the guidelines of the Institutional Animal Care and Use Committee of the Institute for Nutritional Sciences in China. All animal studies were approved by the Animal Ethical and Welfare Committee of Shenzhen University (Permit number: AEWC-20140615-002).

BEOV (Wuhan Biocar Bio-Pharm Company Limited, China) was prepared at a concentration of 200 mg/ml and then diluted with drinking water to the final concentration before use. Starting at 6 months of age, the APPSwe/PS1dE9 mice (*n* = 12, six females and six males) were treated with BEOV, which was dissolved in ultrapure water at a concentration of 68.4 μg/ml (0.2 mmol/L) and 342 μg/ml (1.0 mmol/L) for 90 consecutive days. Equal numbers of female and male APPSwe/PS1dE9 (*n* = 12) and wild-type mice (*n* = 12, as the negative control) were treated with water. Each mouse drank 3–4 ml water per day, the low dose and high dose of which were equivalent to 0.206–0.274 mg and 1.03–1.37 mg BEOV intake per day, respectively. When the animals grew up to 9 months old, all four groups of mice (*n* = 8, four females and four males) were subjected to behavior tests and six mice (*n* = 6, three females and three males) in each group were subjected to ^18^F-labeled fluoro-deoxyglucose positron emission tomography (^18^F-FDG-PET) scanning.

### Cognitive and Behavioral Tests

Three different types of behavioral tests were conducted to assess memory capacity, learning ability, anxiety, and motor skills at the end of the drug treatment. The Morris water maze (MWM) tests were performed in a circular tank (122 cm in diameter and 58 cm high) which was virtually divided in four quadrants. The tank was filled with opaque water, which was maintained at 22°C ± 1°C, and a submerged escape platform (10 cm in diameter) was placed in a constant position 1.0 cm below the water surface in one quadrant. Briefly, four different color visual cues including triangle, circle, square, and rectangle were fixed on the inner wall and the mice were trained to find a submerged escape platform in water maze for five consecutive days. In each training trial, the mice were gently released facing the inner wall into the water at a semi-random start location and allowed to search the platform for 120 s. If the mice found the hidden platform within 120 s, they remained there for 5 s, while the mice that failed to find the platform within 120 s were manually guided to the hidden platform and remained there for 30 s. Twenty-four hours and 72 h after the last training trial, the hidden platform was removed and each mouse was allowed to swim freely for 120 s. The number of times crossing the platform, time staying in the target quadrant, and swimming speed were recorded using the Watermaze MT-200 software (MPEG-4 Image).

Open field test (OFT) was performed 3 days after MWM test. The open field was located in a quiet room and the mice were placed in the center of a 30 × 30 × 20 cm open-top chamber to adapt to the environment. After a 1-min acclimatization period, the mice were allowed to explore the open field for 5 min and behavioral data in the OFT were collected by a video camera. After each trial, the mice were then removed from the chamber and returned to their home cages and the chamber was cleaned with 70% ethanol. The anxiety degree and locomotor activity of the mice were assessed by recording defecation and the number of grid crossings, respectively, within 5 min in the open field.

For step-down passive avoidance test, the equipment consisted of five plastic black chambers and the floor of each chamber consisted of parallel, stainless-steel grids and a well-insulated platform with a height of 2.5 cm, which was positioned in a corner of the chamber. Before the formal experiment session, the mice were gently positioned on the grid floor and each mouse was given 180 s for adaptation and then electric currents (36 V) were delivered for 300 consecutive seconds. The animals received mild electrical shock and immediately performed an instinctive response to jump onto the platform to avoid the electric shock. The error times of each mouse (number of times that the mouse stepped down from platform) and the time of each mouse stayed on the insulated platform were recorded to assess the memory retention.

### ^18^F-FDG-PET

All the PET scans were performed with the Trans-PET^®^ BioCaliburn^®^ 700 (Raycan Technology Company Limited, Suzhou, China) in the PET center of Union Hospital. Briefly, after the overnight fast, the mice were injected with 7.4 MBq ^18^F-fluorodeoxyglucose (^18^F-FDG) under anesthesia in a volume of 100 μl in vein. For static scan, the scan time was 10 min after an uptake period of 60 min. The reconstructed parameters were 12 iterations and 12 subsets. For dynamic scan, the mice were scanned 60 min immediately after the injection. The reconstructed parameters were 1 iteration and 12 subsets, and the whole scanning time was divided into a total of 26 frames: 5 s × 6, 10 s × 3, 30 s × 4, 60 s × 2,120 s × 5,300 s × 3,600 s × 1,300 s × 1, and 900 s × 1. Standard uptake value (SUV mean) of the whole brain, cortex, and hippocampus was quantified by using the software AMIDE.

### Cell Cultures and BEOV Treatment

N2a cells and N2asw cells, stably expressing APP695 with Swedish mutation, were cultured using procedures that were described in detail in previous studies (Jin et al., 2017). Those cells were maintained in a humidified atmosphere with 5% CO_2_ at 37°C. When reaching 80% confluence, they were used for subsequent passage or BEOV treatment. Cell viability was measured by the CCK-8 assay as described previously (Xie et al., 2017).

### Thioflavin-S Staining

Thioflavin-S is a kind of fluorochrome that specifically labels amyloid deposits, and they can be stimulated to produce green fluorescence, which is gently used to detect the amount of Aβ in AD. Briefly, mouse brain sections of 5-μm thickness were deparaffinized and then rehydrated in descending grades of ethanol, while the N2a and N2asw cells were fixed with paraformaldehyde and permeabilized in Triton X-100. Then, the brain sections and N2a/N2asw cells were washed with tris-buffered saline three times, incubated with 0.5% thioflavin-S (Sigma–Aldrich, St. Louis, MO, USA) aqueous solution for 8 min, and immersed in 70% ethanol followed by twice washing with 50% ethanol. Finally, slides were distilled in water three times and cells were washed with tris-buffered saline three times, mounted on glass slides, and examined with a confocal microscope (Olympus, Tokyo, Japan). The plaques were counted in three different fields of view in both the hippocampus and cortex, and six sections from different mice per group were analyzed by using ImageJ software (version 1.51J8, USA).

### Transmission Electron Microscopy (TEM)

The cortex and hippocampus isolated from the mouse brain were fixed with 2.5% glutaraldehyde in 0.1 M phosphate buffer (pH 7.4) at 4°C for 2 h and washed with PBS three times. Then, the samples were coronally sectioned into slices of 100-μm thickness, and the slices were incubated with a fixative containing 1% osmium tetraoxide (OsO_4_) and 1.5% potassium ferrocyanide for 2 h at 4°C. After fixation, the slices were incubated in 2% uranyl acetate overnight at 4°C, dehydrated by sequential treatment in each of 75, 85, 95, and 100% ethanol for 10 min and embedded in epoxy resin. Ultrathin sections were prepared at a 50-nm thickness by using an ultramicrotome (DiATOME knife and Leica ultramicrotome). Finally, the samples were stained with 2% lead citrate and photographed using a FEI Tecnai G2 Spirit TEM. The number of synapses and the thickness of PSD in hippocampus and cortex were measured by using ImageJ software (version 1.51J8, USA).

### Tissue Collection

The mice were deeply anesthetized with isoflurane and sacrificed. Then, the whole brain tissues of each group mice were rapidly removed from the skull and cut sagittally into right and left hemispheres on an ice-cooled board. The cortex and hippocampus of right hemispheres were immediately dissected and removed on ice. The isolated hippocampal and cortical tissue samples were immediately snap frozen in liquid nitrogen and stored at −80°C for further biochemical analysis. Six left hemibrains were postfixed in 4% paraformaldehyde (PFA, Sigma) for 36 h at 4°C and embedded with paraffin and then cut into 5-μm sections for further staining analysis.

### Inductively Coupled Plasma Mass Spectrometry (ICP-MS)

Mouse serum and six left hemispheres were digested with 10% electronic pure nitric acid (Aladdin, Shanghai, China) in the microwave digestion system (CEM-Mars6) at 150°C for 2 h. Digested liquid was collected and diluted with ultrapure water to 20 ml. The concentrations of vanadium (V) in mouse serum and brain of different groups were measured *via* ICP-MS (NexIon 300D, PerkinElmer, Waltham, MA, USA) following the procedures described previously (Day et al., 2017).

### Immunofluorescence Analysis

To detect the expression of IDE and Cathepsin D (CatD), the brain sections (5-μm thickness) were mounted on glass slides. These sections were processed for antigen retrieval with 0.01 mol/L citrate buffer in hyperthermy for 8 min, permeabilized in Triton X-100 for 10 min, and blocked with 5% bovine serum albumin (BSA) in phosphate-buffered saline (PBS) for 30 min. Then, these sections were incubated overnight at 4°C with primary antibody, washed three times with PBS, further incubated for 2 h at 37°C with secondary antibody solution (goat anti-rabbit IgG Alexa Fluor 488; 1:500), and incubated with DAPI (1:5,000 in PBS) for 20 min. These slices were again washed three times with PBS and cover-slipped with vectashield mounting medium. Three equidistant sections were evaluated for each mouse and images were taken by a confocal microscope (Olympus, Tokyo, Japan). The acquired images were quantified with the software Image-Pro Plus Version 6.0 (Media Cybernetics, Bethesda, MD, USA).

### Western Blot Analysis

The hippocampus and cortical tissue samples were homogenized in lysis buffer, 1 mM PMSF, and Protease Inhibitor Cocktail. Protein concentrations were determined by BCA protein assay kit (Thermo Fisher Scientific, Waltham, MA, USA). Tissue samples of equal amounts of proteins (20 μg) were loaded and separated on 12% SDS-PAGE gel and then transferred to polyvinylidene fluoride (PVDF) membranes (Millipore, Kankakee, IL, USA) in the transfer buffer. After transferring, the PVDF membranes were blocked with 5% non-fat dried milk in tris-buffered saline containing 0.1% Tween 20 (TBST) for 2 h at room temperature, washed with TBST for 30 min, and then incubated with corresponding primary antibodies in TBST overnight at 4°C. The primary antibodies we used in this article are listed in Supplementary Table S1. After binding with the corresponding primary antibody, the membranes were washed with TBST for 30 min and incubated with the secondary antibody for another 2 h at room temperature. After three washes in PBST for 30 min, the protein bands were visualized using chemiluminescence with an ECL kit (Pierce), and the band intensities were analyzed using Quantity One software (Bio-Rad, Hercules, CA, USA). GAPDH was detected as an internal loading control, and all proteins were normalized with their corresponding GAPDH levels for presenting the semiquantitative results.

### Determination of Serum Biochemical Parameters

Blood was drawn from the mouse using a capillary tube under anesthesia. The samples were centrifuged at 3,000× *g* for 30 min within 2 h after collection. The levels of aspartate transaminase (AST, an indicator of liver damage), urea, and creatinine (the indicators of kidney damage) were measured using the commercially available kits (Shenzhen Kubel Biotechnology Company Limited, China) on the Imagic-M7 Automatic Biochemical Analyzer (Shenzhen Kubel Biotechnology Company Limited, China) according to the manufacturer’s instructions.

### Statistical Analysis

In this study, all results were obtained from repeated experiments and expressed as means ± standard error of the mean (SEM). Shapiro–Wilk’s test (Villasenor Alva and Estrada, 2009) was applied to check whether the data fit the normal distribution. We found that the data approximately obey Gaussian distribution. Repeated-measures ANOVA was performed for dynamic ^18^F-FDG scanning and behavioral data. Other data were analyzed by using one-way ANOVA with a Tukey’s multiple comparison test. *P* < 0.05 was considered as statistically significant. Statistical analyses were performed using GraphPad Prism 8.0 (GraphPad Software Inc., La Jolla, CA, USA).

## Results

### BEOV Ameliorated Learning and Memory Deficits of the AD Model Mice

The APPSwe/PS1dE9 AD mice did not display neurotoxicity or overt side effects after treatment with 0.2 mmol/L and 1.0 mmol/L BEOV for 3 months, starting at the age of 6 months. No significant alterations were detected in body weight and the levels of serum AST, urea, and creatinine among the APPSwe/PS1dE9 AD mice and BEOV-treated AD mice (Supplementary Figure S1), which indicated no damage to liver and renal function by BEOV at the doses used in this article. The uptake of vanadium was measured in serum and brain and shown in Supplementary Figure S2. Both serum and brain levels of vanadium were significantly promoted by the treatment of BEOV, indicating that vanadium had crossed the blood-brain barrier of APPSwe/PS1dE9 AD mouse.

**Figure 1:**
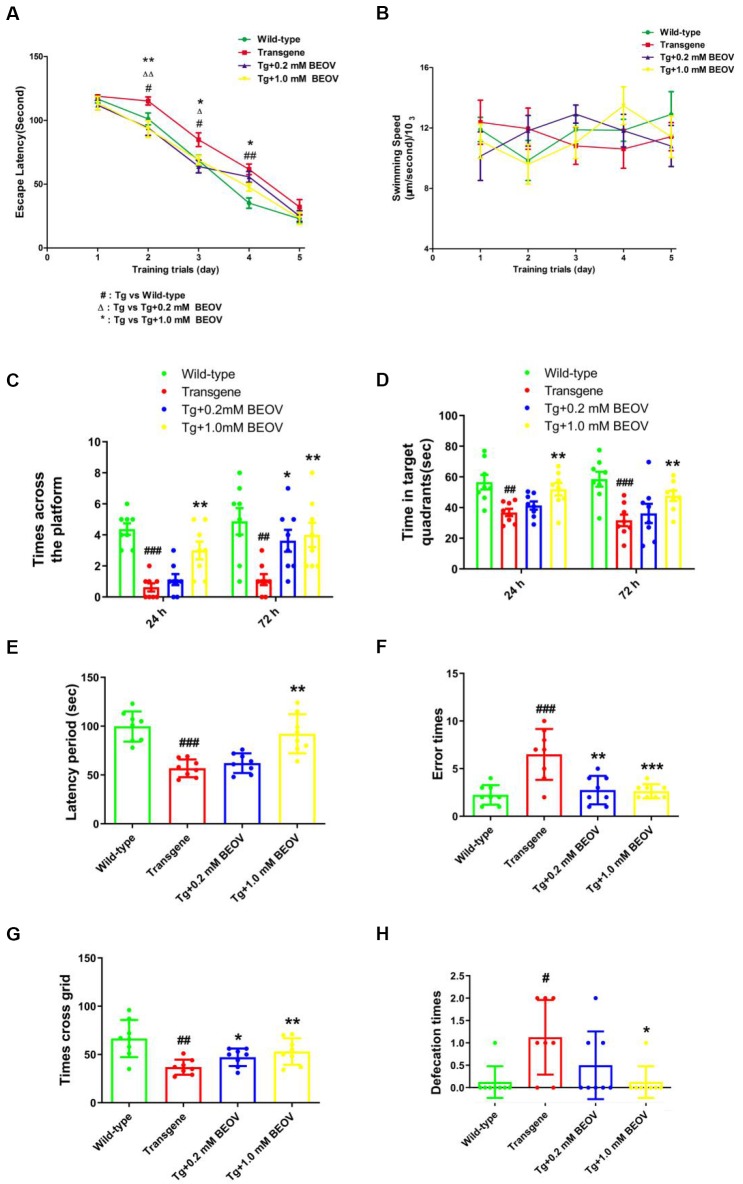
Bis(ethylmaltolato) oxidovanadium (IV) (BEOV) treatment ameliorated learning and memory deficits in APPSwe/PS1dE9 mice. **(A–D)** The Morris water maze (MWM) test was used to assess contextual memory in mice. **(A,B)** During the 5 days of spatial orientation trials, escape latency and swimming speed were measured to evaluate the memory and locomotor ability of the WT, APPSwe/PS1dE9, and BEOV-treated APPSwe/PS1dE9 mice, respectively. **(C,D)** For the 24-h and 72-h probe trials, the number of crossing over the original platform area and the time spent in the target quadrants were recorded. **(E,F)** The learning and memory impairments in APPSwe/PS1dE9 mice were evaluated by step-down avoidance test. **(G,H)** Depression and anxiety-related behavior in APPSwe/PS1dE9 mice were evaluated by open-field test. ^#^WT group vs. Alzheimer’s disease (AD) group; *AD + BEOV group vs. AD group. *n* = 8, four females and four males; ^#^*P* < 0.05, ^##^*P* < 0.01, ^###^*P* < 0.001, *P < 0.05, **P < 0.01, and ***P < 0.001, respectively.

To test whether BEOV treatment had any impact on memory impairment, we subjected 9-month-old WT mice, APPSwe/PS1dE9 AD mice, and BEOV-treated AD mice to the MWM test to evaluate their memory and spatial learning. As expected, APPSwe/PS1dE9 mice showed a significantly longer latency (day 2, day 3, and day 4, *P* < 0.05) to reach the hidden platform than WT mice during the navigation testing. The APPSwe/PS1dE9 mice supplemented with 0.2 mmol/L or 1.0 mmol/L BEOV took less time to find the location of the hidden platform compared with non-treated APPSwe/PS1dE9 mice (Figure 1A), suggesting that BEOV improved the learning deficit of AD mice. No significant differences were found in the motor activity of mice in the four groups (Figure 1B), suggesting that BEOV did not affect the athletic ability of transgenic AD mice. In the probe trial test, 24 and 72 h after the last training on the 5th day, the hidden platform was removed and the four groups of mice were subjected to a probe trial to assess their short-term memory and long-term memory, respectively. Compared with WT mice, the APPSwe/PS1dE9 group mice spent less swimming time in the target quadrant and exhibited decreased number of crossing over the previous platform position, whereas the BEOV-treated AD group had a significantly increased swimming time in the target quadrant and higher frequency of crossing in the position where the platform was previously removed (Figures 1C,D). These results together suggested that BEOV treatment ameliorated learning and memory deficits in APPSwe/PS1dE9 mice.

The step-down passive avoidance test was carried out after the MWM test. The APPSwe/PS1dE9 mice exhibited very poor learning ability and memory showing as higher average number of errors and shorter latency period than the WT group. Treatment with 0.2 and 1.0 mmol/L BEOV remarkably reduced the number of errors and increased the latency period in AD mice (Figures 1E,F).

In order to further explore the effect of BEOV on exploring ability and anxiety of APPSwe/PS1dE9 mice, the OFT was conducted. In novel testing environments, APPSwe/PS1dE9 mice exhibited weaker exploration ability when compared with WT mice and BEOV-treated APPSwe/PS1dE9 mice. APPSwe/PS1dE9 mice exhibited fewer numbers of crossed grids than the WT mice and the BEOV-treated APPSwe/PS1dE9 mice in an open field (a 30 × 30 × 20 cm open-top chamber) within a 5-min test (Figure 1G). Moreover, the number of defecation was also recorded in order to evaluate the degree of anxiety in AD mice. We found that the APPSwe/PS1dE9 mice had a higher degree of anxiety including a larger number of defecation than the WT mice, while 1.0 mmol/L BEOV treatment significantly reduced number of defecation in the AD mice and exhibited decreased degree of anxiety (Figure 1H). These results indicated that BEOV ameliorated learning and memory deficits in APPSwe/PS1dE9 mice.

### BEOV Promoted Glucose Uptake in the Brain of AD Model Mice

**Figure 2:**
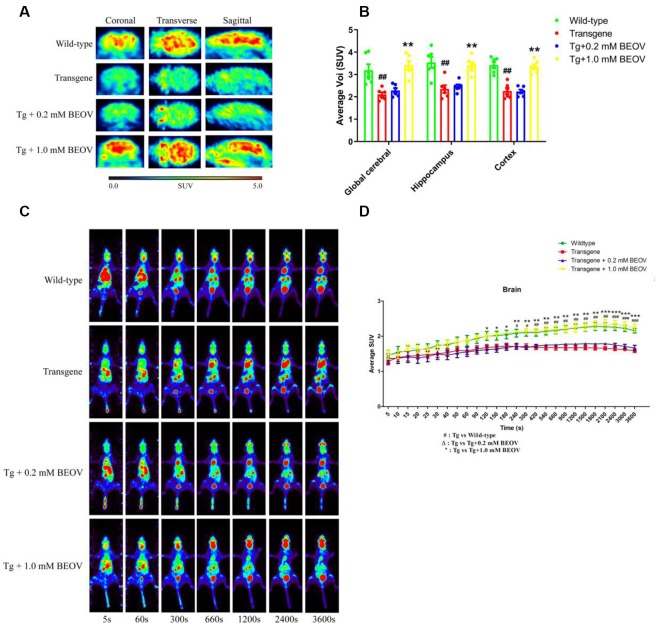
BEOV promoted glucose uptake in the brain of APPSwe/PS1dE9 mice. **(A)** Static imaging of cerebral glucose uptake by the ^18^F-FDG PET in the WT, APPSwe/PS1dE9, and BEOV-treated APPSwe/PS1dE9 mice at the age of 9 months. **(B)** Quantitative analysis of ^18^F-FDG uptake in global cerebral, hippocampus and cortex of the mice. **(C,D)** Dynamic imaging of cerebral glucose uptake and quantitative analysis of ^18^F-FDG uptake levels in mouse brain from 5 to 3,600 s. ^#^WT group vs. AD group; *AD + BEOV group vs. AD group. *n* = 6, three females and three males; ^#^*P* < 0.05, ^##^*P* < 0.01, ^###^*P* < 0.001, *P < 0.05, **P < 0.01, and ***P < 0.001, respectively.

Cerebral glucose uptake reflects the vitality of mouse brain under pathophysiolgical conditions. In this study, ^18^F-FDG PET was conducted to detect the cerebral glucose metabolism *in vivo* in WT, APPSwe/PS1dE9, and BEOV-treated APPSwe/PS1dE9 mice. Typical PET images of ^18^F-FDG PET static scanning are shown in Figure 2A. Compared with WT mice, AD mice showed an obvious deficiency in glucose utilization in three brain regions, including the global cerebral, hippocampus, and cortex. BEOV (1.0 mmol/L) treatment significantly reversed the glucose uptake defects in the AD group, while no significant difference was found between AD and the 0.2 mmol/L BEOV-treated group (Figure 2B). ^18^F-FDG PET dynamic scanning was carried out 7 days after static scanning and results indicated that the uptake level of ^18^F-FDG in the brain of the four mouse groups was increased over the time period of 60 min (Figure 2C and Supplementary Videos S1–S4). In the beginning, there was no difference of ^18^F-FDG brain uptake among the WT, AD, and BEOV-treated AD groups. At 120 and 240 s, the increased uptake levels of ^18^F-FDG appeared in the brain of the WT and 1.0 mmol/L BEOV-treated AD group when compared with the AD group. No significant change appeared in the brain of the 0.2 mmol/L BEOV-treated AD group from 0 to 3,600 s (Figure 2D). In addition, treatment with BEOV for 3 months suppressed the reduction of cerebral glucose metabolism in the APPSwe/PS1dE9 mice.

### BEOV Protected Hippocampal and Cortical Synapses of AD Model Mice

**Figure 3:**
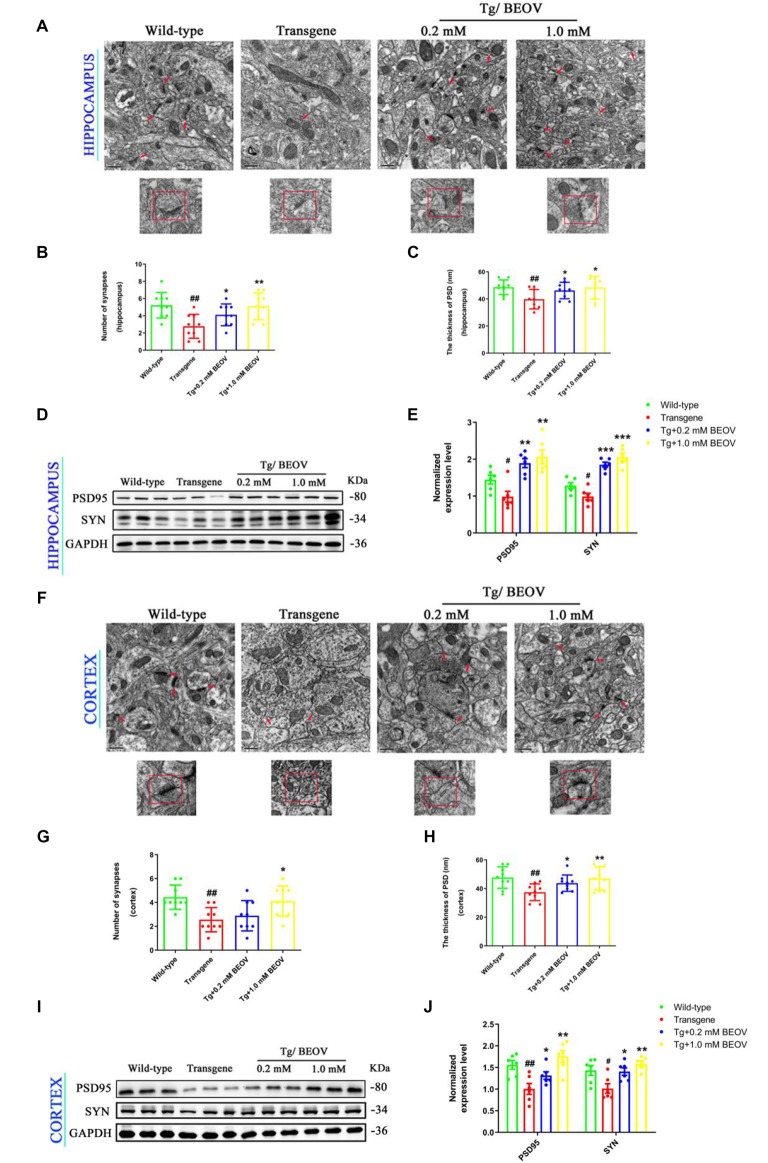
BEOV protected hippocampal and cortical synapses in APPSwe/PS1dE9 mice. **(A,F)** Synapses in the cornu ammonis 3 (CA3) region of the hippocampus and the layer V region of cortex of WT, APPSWE/PS1DE9, and BEOV-treated APPSwe/PS1dE9 mice were imaged by transmission electron microscopy (TEM). Scale bar = 0.5 μm. **(B,G)** Quantitative graph of the numbers of synapses in equal areas. The numbers of synapses were counted in three fields of view on each ultrathin section, and three ultrathin sections from different mice in each group were analyzed. **(C,H)** Quantitative analysis of the thickness of postsynaptic density (PSD) in each group. ^#^WT vs. AD; *AD + BEOV vs. AD. *n* = 3, ^#^*P* < 0.05, ^##^*P* < 0.01, *P < 0.05, and **P < 0.01, respectively. **(D,I)** and **(E,J)** Western blot analyses and semi-quantification of proteins containing synaptophysin (SYN) and postsynaptic protein (PSD95) in the hippocampus and cortex of mice at the age of 9 months. Quantitative results were normalized against the levels of GAPDH. ^#^WT group vs. AD group; *AD + BEOV group vs. AD group. *n* = 6, three females and three males; ^#^*P* < 0.05, ^##^*P* < 0.01, *P < 0.05, **P < 0.01, and ***P < 0.001, respectively.

Recent *in vivo* studies have shown that synaptic deficits and neuronal loss appeared in the hippocampus and cortex of AD mice (Ma et al., 2017; Sun et al., 2017). In order to study the underlying mechanisms of improved memory and learning ability in APPSwe/PS1dE9 mice, the effect of BEOV on synapses was investigated by TEM. Impairment and loss of synapses were found in the hippocampus of APPSwe/PS1dE9 mice compared to the WT mice. After treatment with 0.2 mmol/L and 1.0 mmol/L BEOV for 3 months, the decreased number of synapses and thickness of postsynaptic density (PSD) were suppressed in the cornu ammonis 3 (CA3) region of the hippocampus (Figures 3A–C). The expression levels of synapse-related proteins including synaptophysin (SYN) and postsynaptic protein (PSD95) were also analyzed in the hippocampus by Western blot. Levels of SYN and PSD95 were significantly decreased in APPSwe/PS1dE9 mice compared to WT mice, while BEOV treatment remarkably increased their protein expression levels in AD mice (Figures 3D,E). TEM and Western blot analysis were also used to investigate the role of BEOV in cortical synapses. As shown in Figures 3F–J, BEOV significantly increased the number of synapses and the levels of some synaptic proteins, and it also improved synaptic damage in the cortex of APPSwe/PS1dE9 mice.

Activation of c-Jun N-terminal kinase (JNK) has been reported in APPSwe/PS1dE9 mice to induce long-term synaptic toxicity and cognition impairment (Fang et al., 2017; Liang et al., 2019). Vanadium was demonstrated to decrease phosphorylation of JNK (p-JNK) and thus reduce JNK activation (Wang et al., 2015; Kim and Kim, 2019). To further evaluate the role of BEOV on decreased synapses, JNK expression and phosphorylated JNK levels were analyzed in both hippocampus and cortex of APPSwe/PS1dE9 mice. As shown in Supplementary Figures S3A–D, BEOV significantly decreased the ratio of p-JNK/JNK in both hippocampus and cortex, which indicated that BEOV could improve synaptic damage possibly through downgrading the activation of JNK.

### BEOV Reduced Aβ Generation in N2asw Cells and AD Model Mice

**Figure 4:**
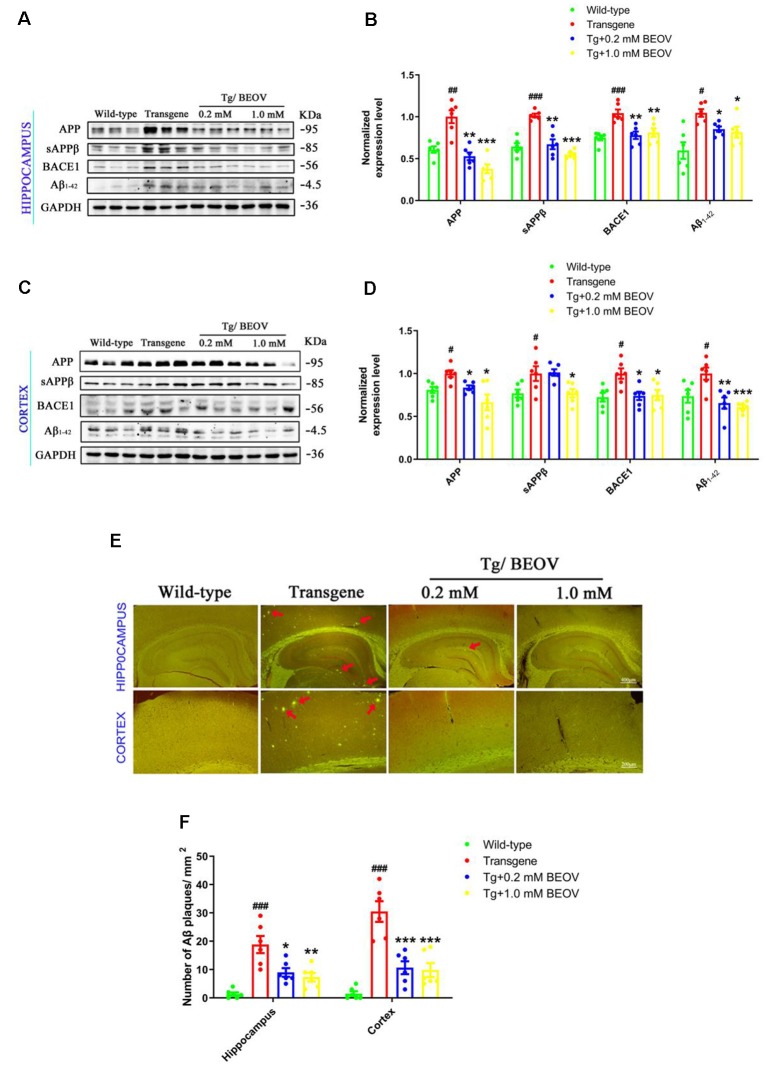
BEOV reduced Aβ generation in APPSwe/PS1dE9 mice. **(A–D)** Western blot analyses and semi-quantification of several key proteins involved in amyloidogenic cascade in the hippocampus and cortex of WT, APPSwe/PS1dE9, and BEOV-treated APPSwe/PS1dE9 mice. Quantitative results were normalized against the levels of GAPDH. **(E)** Thioflavin S staining of Aβ plaques in the brains of 9-month-old mice. Scale bar: 200 or 400 μm. **(F)** Quantification of the number of Aβ plaques/mm^2^. The plaques were counted in three different fields of view in both hippocampus and cortex, and six sections from different mice in each group were analyzed. ^#^WT group vs. AD group; *AD + BEOV group vs. AD group. *n* = 6, three females and three males; ^#^*P* < 0.05, ^##^*P* < 0.01, ^###^*P* < 0.001, *P < 0.05, **P < 0.01, and ***P < 0.001, respectively.

Aβ burden in hippocampus and cortex is a typical feature of AD. Aβ is generated generally through two-step proteolytic processing of the APP: cleavage of APP by β-site APP cleaving enzyme 1 (BACE1) generates a β-C-terminal fragment (β-CTF), which is further cut by γ-secretase and yields Aβ. Alternative cleavage of APP by α-secretases, mainly ADAM10, within the Aβ domain precludes Aβ production (Haass et al., 2012). In order to study the effects of BEOV on the processing of Aβ generation in 9-month-old APPSwe/PS1dE9 mice, we measured the expression of APP, sAPPβ, BACE1, and Aβ_1–42_ in hippocampus and cortex by Western blot analysis. We found that the protein levels of APP, BACE1, sAPPb, and Aβ_1–42_ were significantly increased in both hippocampus and cortex of AD mouse when compared with those in WT mice. Treatment with BEOV in the AD mouse suppressed the levels of APP, BACE1, sAPPβ, and Aβ_1–42_ remarkably (Figures 4A–D). Besides, we also used thioflavin S to stain the brain slices of the four mouse groups. The fluorescent images showed the inhibitory effect of BEOV on Aβ deposits in the cortex and hippocampus of APPSwe/PS1dE9 mice after 3-month treatment, which were consistent with the Western blot results (Figures 4E,F).

**Figure 5:**
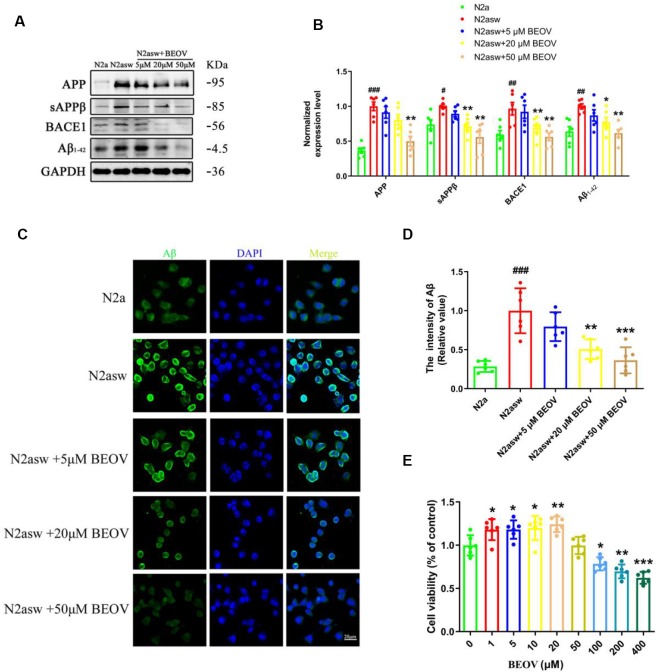
Inhibition of BEOV on the amyloidogenic cascade in N2asw cells. **(A,B)** Western blot analysis and semi-quantification of several key proteins involved in the amyloidogenic cascade in N2a, N2asw, and BEOV-treated N2asw cells. Quantitative results were normalized against the levels of GAPDH. **(C)** Thioflavin S staining of Aβ in N2a, N2asw, and BEOV-treated N2asw cells. **(D)** The staining intensity of Aβ in those cells was quantified by software Image-Pro Plus Version 6.0. **(E)** CCK-8 assay of the viability of N2asw cells treated with BEOV at a series of dosages (1–400 μM). Scale bar: 20 μm. ^#^N2a group vs. N2asw group; *N2asw + BEOV group vs. N2asw group. *n* = 6 wells per group, ^#^*P* < 0.05, ^##^*P* < 0.01, ^###^*P* < 0.001, *P < 0.05, **P < 0.01, and ***P < 0.001, respectively.

To further evaluate the role of BEOV on Aβ generation, the AD model N2asw cells were treated with BEOV at concentrations of 0, 5, 20, and 50 μmol/L and N2a cells were used as the control. The expression levels of APP, sAPPβ, BACE1, and Aβ_1–42_ were analyzed by Western blotting. As shown in Figures 5A,B, BEOV remarkably reduced the levels of APP, BACE1, sAPPβ, and Aβ_1–42_ in N2asw cells. Similar results were also illustrated by thioflavin S staining (Figures 5C,D). The concentrations of BEOV in N2asw cell treatment were based on the result of cell viability measurement (Figure 5E). Therefore, BEOV could reduce Aβ deposition by downgrading the levels of APP, BACE1, and sAPPβ.

### BEOV Up-Regulated the Expression of PPARγ and IDE in the AD Mice

**Figure 6:**
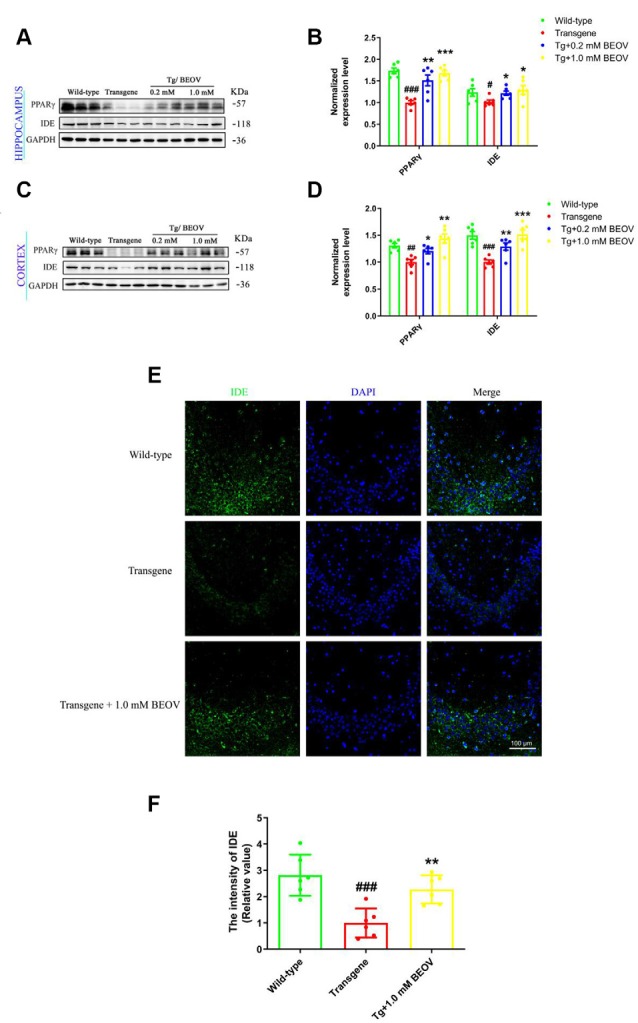
BEOV up-regulated the expression of Peroxisome proliferator-activated receptor gamma (PPARγ) and insulin-degrading enzyme (IDE) in APPSwe/PS1dE9 mice. **(A,C)** Western blot analyses of PPARγ and IDE levels in the hippocampus and cortex of WT, APPSwe/PS1dE9, and BEOV-treated APPSwe/PS1dE9 mice. **(B,D)** Semi-quantification of PPARγ and IDE in the hippocampus and cortex of 9-month-old mice. Quantitative results were normalized against the levels of GAPDH. **(E)** Representative immunofluorescent images of IDE expression in the CA3 region of the hippocampus. **(F)** The staining intensity of IDE was quantified and six sections from different mice in each group were analyzed. Scale bar: 100 μm. ^#^WT group vs. AD group; *AD + BEOV group vs. AD group. *n* = 6, three females and three males; ^#^*P* < 0.05, ^##^*P* < 0.01, ^###^*P* < 0.001, *P < 0.05, **P < 0.01, and ***P < 0.001, respectively.

PPARγ is a transcription factor that is present in the BACE1 promoter and reduces BACE1 activity (Chen et al., 2012) and transcriptionally regulates the expression of IDE in primary neurons (Du et al., 2009). Some studies have also demonstrated that IDE could reduce Aβ toxicity and promote Aβ clearance (Huang et al., 2019) as well as reduce the level of IDE or genetic mutation of IDE-altered AD pathology (Cook et al., 2003; Prince et al., 2003). To elucidate the mechanism of BEOV in inhibiting Aβ production and promoting Aβ clearance in the brain of APPSwe/PS1dE9 mice, the expression levels of PPARγ and IDE were assessed by Western blot analyses. As shown in Figures 6A,B, the levels of PPARγ and IDE in the hippocampus of AD mice were lower than the WT group, while mice treated with 0.2 and 1.0 mmol/L BEOV significantly increased their protein expression. Increased PPARγ and IDE levels were also found in the cortex of BEOV-treated AD mice (Figures 6C,D). Similar results were also illustrated in the hippocampus by immunofluorescence analysis (Figures 6E,F). The results indicated that BEOV might reduce Aβ generation and deposition by up-regulating the expression of PPARγ and IDE.

### BEOV Down-Regulated PTP1B Activity and Its Downstream Pathways in the AD Model Mice

**Figure 7:**
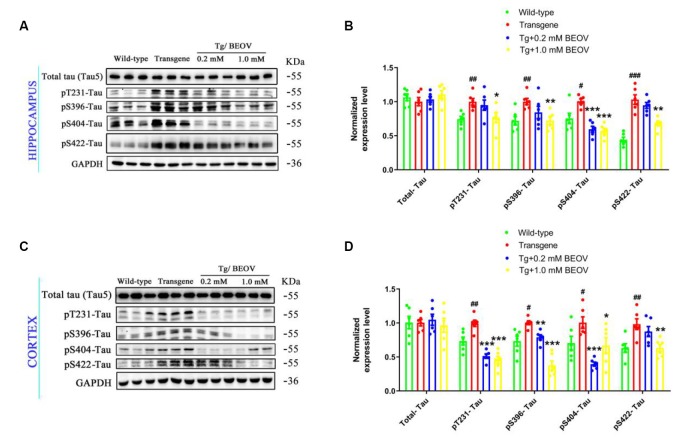
BEOV inhibited tau hyperphosphorylation in APPSwe/PS1dE9 mice. **(A,C)** The level of total tau and phosphorylated tau at Thr231, Ser396, Ser404, and Ser422 were detected using Western blot both in the hippocampus and cortex of WT, APPSwe/PS1dE9, and BEOV-treated APPSwe/PS1dE9 mice. **(B,D)** Semi-quantification of total tau and phosphorylated tau at different amino acid positions. Quantitative results of phosphorylated tau were normalized against the levels of total tau (tau5). ^#^WT group vs. AD group; *AD + BEOV group vs. AD group. *n* = 6, three females and three males; ^#^*P* < 0.05, ^##^*P* < 0.01, ^###^*P* < 0.001, *P < 0.05, **P < 0.01, and ***P < 0.001, respectively.

Hyperphosphorylated tau is a typical feature of AD and plays an important role in the pathogenesis of this disease (Zhang et al., 2014). Increased levels of phosphorylated tau have been reported previously in APPSwe/PS1dE9 mice (Ramos-Rodriguez et al., 2013; Wei et al., 2016; Zhang et al., 2016). In this article, the levels of phosphorylated tau were found remarkably increased in the hippocampus and the total tau protein (tau5) was not significantly changed compared with the WT group (Figures 7A,B). Treatment with 0.2 mmol/L or 1.0 mmol/L BEOV did not affect the expression level of total tau while it significantly inhibited tau phosphorylation at T231, S396, S404, and S422 sites (pT231-tau, pS396-tau, pS422-tau, and pS404-tau, respectively). Resembling the results in the hippocampus, BEOV also decreased the level of tau phosphorylation in the cortex of APPSwe/PS1dE9 mice (Figures 7C,D).

**Figure 8:**
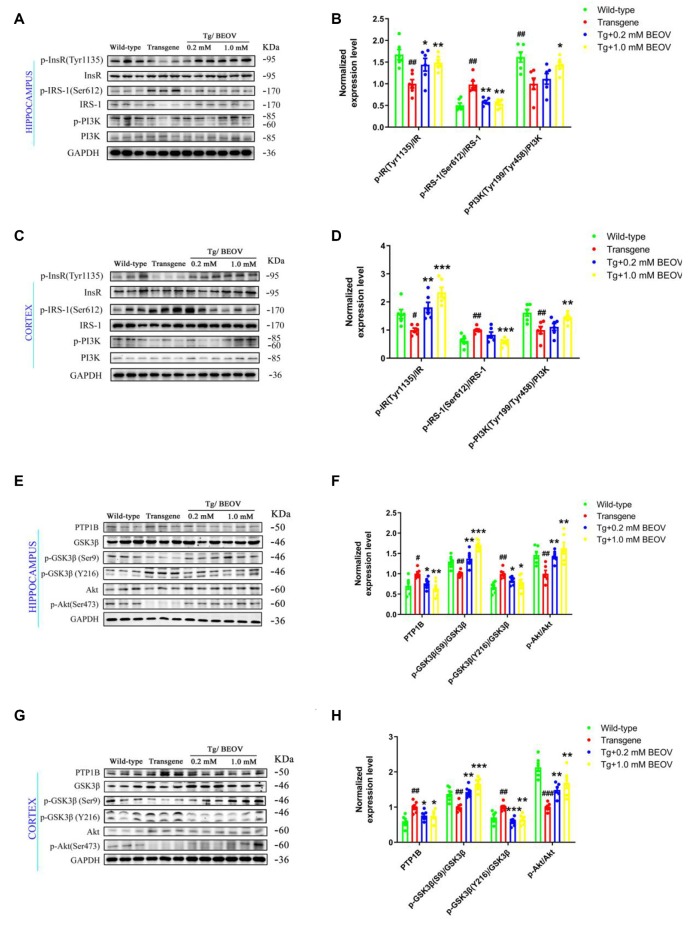
Effect of BEOV on PTP1B activity, insulin signaling and tau hyperphosphorylation in APPswe/PS1dE9 mice. **(A,C,E,G)** Western blotting analyses of several key proteins involved in the pathway of tau phosphorylation in mouse hippocampus and cortex. **(B,D,F,H)** Semi-quantification of PTP1B, the relative ratio of p-IR/IR, p-IRS-1/IRS-1, p-PI3K/PI3K, pS9-GSK3β/GSK3β, pY216-GSK3β/GSK3β, and p-Akt/Akt in the hippocampus and cortex of WT, APPSwe/PS1dE9, and BEOV-treated APPSwe/PS1dE9 mice. Quantitative results were normalized against the levels of GAPDH. ^#^WT group vs. AD group; *AD + BEOV group vs. AD group. *n* = 6, three females and three males; ^#^*P* < 0.05, ^##^*P* < 0.01, ^###^*P* < 0.001, *P < 0.05, **P < 0.01, and ***P < 0.001, respectively.

PTP1B is known as a negative regulator of insulin signaling. The protein level and activity of PTP1B were associated with the signal transduction of insulin receptor (InR) and insulin receptor substrate (IRS), which is a key pathway in activating the PI3K-Akt pathway (Kenner et al., 1996; Zinker et al., 2002; Ropelle et al., 2006; Kanno et al., 2016). Serine phosphorylation of IRS proteins is believed to be a major mechanism of suppression of IRS-1 activity that contributes to insulin resistance (Ropelle et al., 2006). Glycogen synthase kinase3β (GSK3β) and protein kinase B (Akt) are two key enzymes that play critical roles in regulating tau hyperphosphorylation and dephosphorylation. Hyperphosphorylated tau could be inhibited by regulating the IRS-1/PI3K/Akt/GSK-3β pathway in AD rats (Xiong et al., 2020). In order to elucidate the mechanism of BEOV in inhibiting tau hyperphosphorylation in the brain of APPSwe/PS1dE9 mice, we assessed the expression levels of PTP1B, IR, IRS-1, PI3K, Akt, GSK-3β, and their corresponding phosphorylated forms at specific sites. As shown in Figure 8, the expression level of PTP1B in APPSwe/PS1dE9 mice was much higher than that in the WT group, and BEOV treatment significantly reduced the level of PTP1B in the cortex and hippocampus of APPSwe/PS1dE9 mice. It has been reported that IRS-1 activity was inhibited by phosphorylation at Ser612 (pS612). GSK3β activity was inhibited by phosphorylation at S9 and increased by phosphorylation at Tyr216 (pY216), respectively. Meanwhile, there was a positive correlation between the activity of Akt and the ratio of p-Akt/Akt, or the activity of PI3K and the ratio of p-PI3K/PI3K. We found that the ratios of pY1135-IR/IR, pY199&pY458-PI3K/PI3K, pS9-GSK3β/GSK3β, and pS473-Akt/Akt were remarkably decreased and pS612-IRS-1/IRS-1 and pY216-GSK3β/GSK3β were significantly increased in both the cortex and hippocampus of APPSwe/PS1dE9 mice when compared with WT mice. BEOV treatment significantly increased the ratios of pY1135-IR/IR, pY199&pY458-PI3K/PI3K, pS9-GSK3β/GSK3β, and pS473-Akt/Akt, and reduced the ratios of pS612-IRS-1/IRS-1 and pY216-GSK3β/GSK3β (Figure 8), leading to the inhibitory effect on tau hyperphosphorylation in APPSwe/PS1dE9 mice.

### BEOV Influenced the Autophagic Process in AD Mice

**Figure 9:**
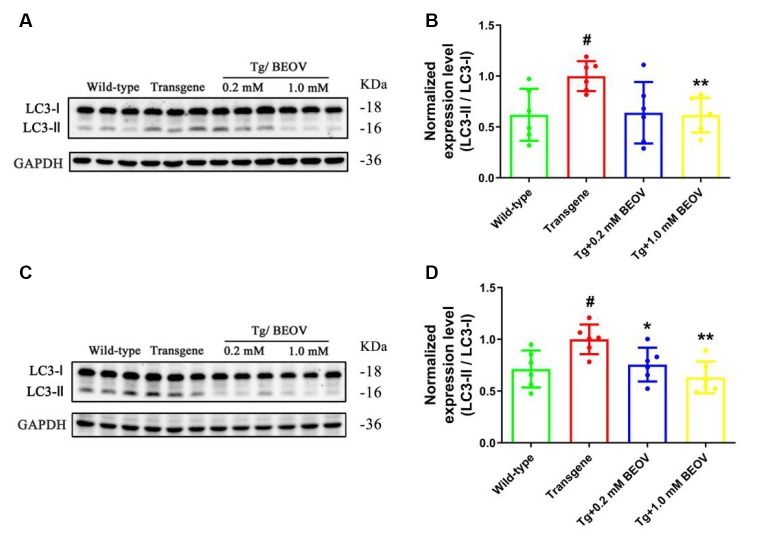
The LC3-II/LC3-I ratio was decreased by BEOV in both the hippocampus and cortex of APPSwe/PS1dE9 mice. **(A,C)** Western blotting analyses of the LC3-I and LC3-II levels in the hippocampus and cortex of WT, APPSwe/PS1dE9, and BEOV-treated APPSwe/PS1dE9 mice. **(B,D)** Semi-quantification of the relative ratio of LC3-II/LC3-I in mouse hippocampus and cortex. Quantitative results were normalized against the levels of GAPDH. ^#^WT group vs. AD group; *AD + BEOV group vs. AD group. *n* = 6, three females and three males; ^#^*P* < 0.05, *P < 0.05 and **P < 0.01, respectively.

It has been reported that autophagy could affect the clearance of Aβ and hyperphosphorylated tau (Zhang et al., 2017; Song et al., 2018). The effect of BEOV treatment on autophagy was thus explored to investigate the mechanism of BEOV in reducing hyperphosphorylated tau and Aβ aggregates. Light chain protein 3 (LC3) is generally used as a marker of autophagy and the full-length LC3 (LC3-I) is converted to its cleaved form LC3-II during the formation of autophagosome (Noboru and Tamotsu, 2007). We detected the ratio of LC3-II/LC3-I in the brain by Western blot analyses. As shown in Figure 9, there is a remarkable increase in the ratio of LC3-II/LC3-I in APPSwe/PS1dE9 mice compared with WT mice, while the ratio of LC3-II/LC3-I was significantly decreased in both the hippocampus and cortex after treatment with BEOV. Thus, BEOV decreased the formation of autophagosomes.

**Figure 10:**
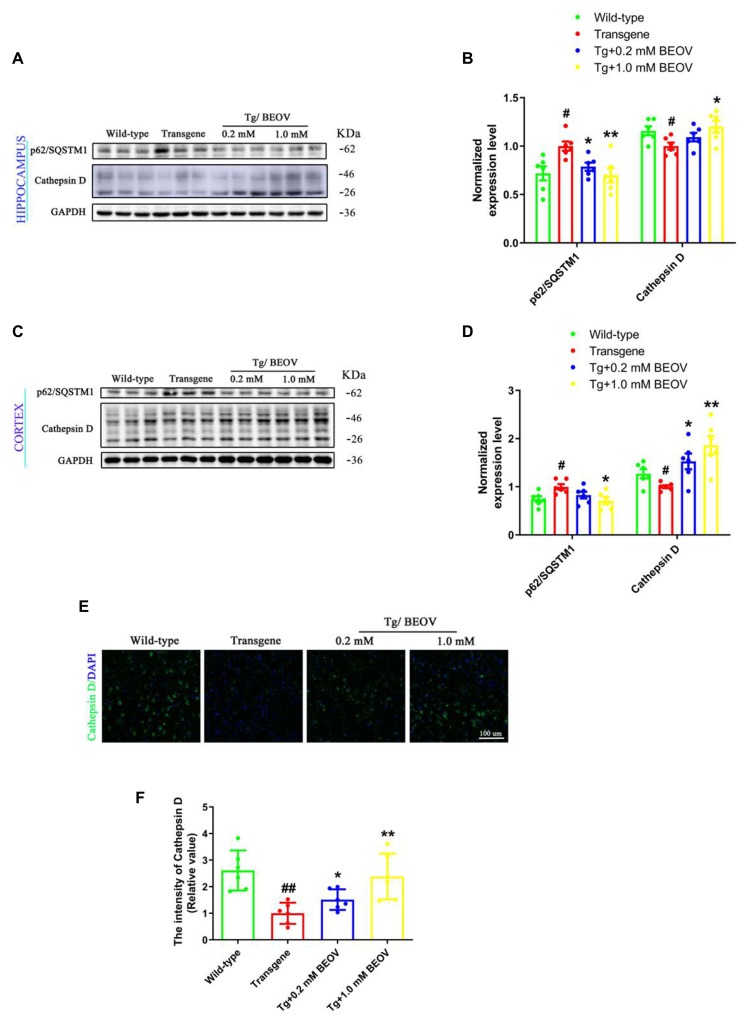
BEOV reduced the p62 levels and increased the Cathepsin D levels. **(A,C)** Western blot analyses of p62/SQSTM1 and Cathepsin D levels in hippocampus and cortex of WT, APPSwe/PS1dE9, and BEOV-treated APPSwe/PS1dE9 mice. **(B,D)** Semi-quantification of p62/SQSTM1 and Cathepsin D in mouse hippocampus and cortex. Quantitative results were normalized against the levels of GAPDH. **(E)** Representative immunofluorescent images of Cathepsin D expression in the cortex. Scale bar: 100 μm. **(F)** The staining intensity of Cathepsin D was quantified and six sections from different mice in each group were analyzed. ^#^WT group vs. AD group; *AD + BEOV group vs. AD group. *n* = 6, three females and three males; ^#^*P* < 0.05, ^##^*P* < 0.01, *P < 0.05 and **P < 0.001, respectively.

Studies have shown that defects in autophagosome maturation at the stage of autophagosome–lysosome fusion is an important feature of AD pathology (Komatsu et al., 2006). The protein of p62/SQSTM1 (sequestosome 1), a key cargo adaptor protein involved in autophagy–lysosome degradation, is degraded upon autophagosome–lysosome fusion (Xu et al., 2014) and CatD, a lysosomal aspartyl endopeptidase, is generally considered as a “house-keeping enzyme” involved in the clearance of unwanted proteins in AD (Papassotiropoulos et al., 2000; Radisky, 2010). To ascertain whether BEOV can promote autophagic maturation and affect the autophagic clearance of proteins, we detected the levels of p62/SQSTM1 and lysosomal protease CatD in the hippocampus and cortex of WT, APPSwe/PS1dE9, and BEOV-treated APPSwe/PS1dE9 mice. As shown in the Western blot analysis (Figures 10A–D), the expression level of p62/SQSTM1 was remarkably reduced in both the hippocampus and cortex of BEOV-treated APPSwe/PS1dE9 mice. The precursor CatD is activated by proteolysis in the acidified lysosome to produce a mature proteolytic product (Avrahami et al., 2013). The CatD level was significantly decreased in the brain of APPSwe/PS1dE9 mice compared with the WT group, while BEOV treatment significantly increased the expression levels of CatD in both the hippocampus and cortex of AD mice. Similar results were also illustrated in the cortex by immunofluorescence analysis (Figures 10E,F). Therefore, BEOV could enhance the fusion of autophagosomes and lysosomes to promote autophagic degradation.

## Discussion

It is increasingly accepted that T2DM and AD share several common abnormalities including oxidative stress, reduced glucose metabolism, increased insulin resistance, amyloid oligomer toxicity, and impaired cognitive function (Zhao and Townsend, 2009; Tang et al., 2018). Vanadium has been recognized as a biological trace element, and it is currently investigated for the possibility to treat cancer and diabetes (Poucheret et al., 1998; Bishayee et al., 2010). Evidence from recent research also indicate that vanadium plays an important role in protecting against cognitive decline in AD animal models (Dong et al., 2019). BEOV has lower toxicity and higher bioavailability in comparison with inorganic vanadium compounds. Thus, it was selected for the treatment of AD pathology in APPSwe/PS1dE9 mice. In this study, 6-month-old APPSwe/PS1dE9 mice were treated with 0.2 and 1.0 mmol/L BEOV for 3 months, and the results demonstrated that BEOV could reduce Aβ generation and accumulation by up-regulating PPARγ and IDE and inhibiting BACE1 activity. BEOV also down-regulated PTP1B to mediate the IRS-1/PI3K/Akt/GSK3β pathway and thus reduce tau hyperphosphorylation in APPSwe/PS1dE9 mice. In addition, BEOV could regulate the autophagic process to initiate the clearance of Aβ aggregates and hyperphosphorylated tau. Eventually, BEOV demonstrated an increase of cerebral glucose uptake and decreases of synaptic deficit and cognitive impairment.

Aβ cascade is one of the most important pathogenic mechanisms of AD (Small et al., 2001; Palop and Mucke, 2010). PPARγ is activated by vanadium compounds and activated PPARγ is capable of decreasing the Aβ level by inhibiting BACE1 expression and promoting IDE expression (Du et al., 2009; Zhao and Yang, 2013; Cao et al., 2016). Significantly increased plasma Aβ has also been reported in AD when IDE expression was suppressed (Kim et al., 2007). Increased IDE was strongly associated with the decreased levels of Aβ in AD (Du et al., 2009). Down-regulation of both BACE1 and Aβ levels has been considered as an important strategy for anti-AD therapy (Zhu et al., 2013). In this study, after treatment with BEOV for 3 months in APPSwe/PS1dE9 mice, the levels of some key proteins in the amyloidogenic cascade, including APP, sAPPβ, BACE1, Aβ_1–42_, and the amount of Aβ plaques, were significantly decreased in hippocampus and cortex, while the levels of PPARγ and IDE were remarkably increased. Decreased levels of these proteins were also found in the BEOV-treated N2asw cells. These results suggest that BEOV can alleviate AD pathology by reducing Aβ generation, possibly through the increase of PPARγ and IDE and the inhibition of BACE1 activity.

Many studies have reported that tau hyperphosphorylation can be triggered by Aβ_1–42_, and increased levels of abnormally phosphorylated tau are found in APPSwe/PS1dE9 mice (Ott et al., 2011; Ramos-Rodriguez et al., 2013; Wei et al., 2016; Zhang et al., 2016). Tau is abundant in neuronal axons for regulating microtubule (MT) polymerization and stabilizing MT. Under pathological conditions, tau is hyperphosphorylated and separated from MTs, and intraneuronal aggregation of hyperphosphorylated tau constitutes a major neuropathological hallmark of AD (Duan et al., 2012). In this article, the levels of abnormally phosphorylated tau were found significantly increased in APPSwe/PS1dE9 mice and BEOV remarkably reduced the hyperphosphorylation of tau in both the hippocampus and cortex of 9-month-old APPSwe/PS1dE9 mice. An imbalance in protein phosphatases and kinases can directly lead to tau hyperphosphorylation in AD. Previous studies indicate that the serine/threonine protein kinase Akt and GSK3β are two key enzymes that play critical roles for tau phosphorylation (Ali and Kim, 2015). Importantly, GSK3β is inhibited by phosphorylation at S9 and activated by phosphorylation at Y216. Activated GSK3β contributes to tau phosphorylation. Here, BEOV treatment significantly increased the activity of Akt and decreased the activity of GSK-3β, thus reducing tau hyperphosphorylation in both the hippocampus and cortex of APPSwe/PS1dE9 mice. PTP1B is known as a negative regulator of insulin signaling by dephosphorylating IR at Y1135 and PI3K at Y199/Y458, thus inactivating the IR/IRS-1/PI3K-Akt pathway (Kenner et al., 1996; Zinker et al., 2002; Ropelle et al., 2006; Kanno et al., 2016). Serine phosphorylation is a major mechanism in suppressing IRS-1 activity that contributes to insulin resistance (Ropelle et al., 2006). Vanadium compounds have been reported to enhance IR/IRS-1/PI3K/Akt signaling through inhibiting PTP1B in differentiated rat adipocytes (Wu et al., 2013). Consistently, our results in this study also showed that BEOV could decrease PTP1B levels and thus increase the IR/IRS-1/PI3K/Akt signaling, leading to GSK3β inactivation and ultimately reduced tau hyperphosphorylation in APPSwe/PS1dE9 mice.

Autophagy is an essential process and is responsible for the cellular clearance of misfolded and aggregated proteins through autophagolysosomal fusion (Mizushima et al., 2010). Autophagy is also important for neuronal homeostasis, and its dysfunction has been directly linked to the pathological process of neurodegenerative disease such as AD (Boland et al., 2008). Autophagy starts with the formation of autophagosomes, followed by the fusion of these vesicles with lysosomes and their subsequent degradation by enzymes in the lysosome (Mizushima, 2007). As an important multi-functional adaptor molecule, p62/SQSTM1 was incorporated into autophagosomes by direct binding to LC3 and linked ubiquitinated proteins and was finally degraded *via* autophagy (Lippai and Low, 2014). Therefore, p62/SQSTM1 levels are inversely related to autophagic flux. Autophagosomes are marked by LC3-II, and the lysosomal proteolytic enzyme CatD is essential to neuron homeostasis and involved in the degradation of misfolded and aggregated proteins delivered to lysosomes by autophagy. Studies have reported that the protein expression level of CatD is decreased in AD (Urbanelli et al., 2008), and increased CatD activity and expression promoted the fusion of autophagosomes with lysosomes and ultimately contributed to the clearance and degradation of Aβ aggregates and phosphorylated tau (Zhang et al., 2017; Song et al., 2018). In this work, BEOV significantly reduced the ratio of LC3-II/LC3-I in both the hippocampus and cortex of APPSwe/PS1dE9 mice, indicating a decrease of autophagosome formation. On the other hand, BEOV significantly decreased p62/SQSTM1 expression and increased CatD expression, indicating an increase of autophagolysosomal fusion. Taken together, these data suggested that BEOV could inhibit the initiation of autophagy, promote autophagolysosomal fusion, and improve autophagy flux, thus increasing the clearance and degradation of abnormal Aβ and phosphorylated tau in APPSwe/PS1dE9 mice. Additional studies are required to further investigate the influence and mechanism of BEOV on this process.

Multiple studies indicate that Aβ aggregates and hyperphosphorylated tau are two important inducers of synaptic deficits, which is most likely the basis of cognitive impairments in AD (Callahan and Coleman, 1995; Selkoe, 2008). The synaptic proteins including PSD95 and SYN are localized in or associated with presynaptic vesicles, and essential for normal synaptic functions (Elferink and Scheller, 1993; Shin, 2014). Vanadium compounds have been reported to ameliorate cognitive deficits associated with cholinergic neuron degeneration in AD (Dong et al., 2019). In this study, we demonstrated that BEOV increased PSD95 and SYN expression in both the hippocampus and cortex of APPSwe/PS1dE9 mice and improved cognitive impairments. Thus, BEOV is possible to alleviate the behavioral changes of AD mice by improving synaptic deficit.

**Figure 11:**
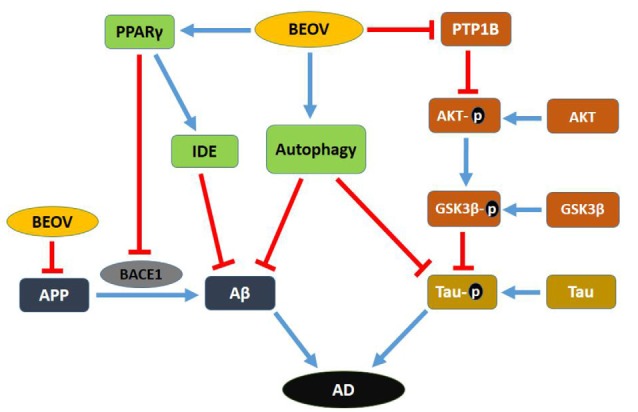
Schematic map of the potential mechanism for the preventive role of BEOV in AD pathology.

In summary, this research used the double transgenic mice APPSwe/PS1dE9 to investigate the effect and mechanism of BEOV on AD pathology. The underlying mechanism of BEOV is summarized in Figure 11. BEOV supplemented to the AD model mice at doses of 0.2 and 1.0 mmol/L reduced Aβ production by promoting PPARγ and IDE expression and inhibiting BACE1 expression. BEOV also reduced tau hyperphosphorylation possibly by inhibiting PTP1B expression and regulating the IR/IRS-1/PI3K/Akt/GSK3β pathway. In addition, BEOV could promote autophagolysosomal fusion and restored autophagic flux to increase the degradation and clearance of Aβ deposits and phosphorylated tau in the brains of APPSwe/PS1dE9 mice. Accordingly, BEOV rescued spatial learning and memory impairments in the AD model mice by improving synaptic deficit. Taken together, although further studies will be necessary, our findings shed new light on the mechanism underlying the biological effects of BEOV on AD.

## Limitations

There were some limitations to our study. First, we only investigated the inhibitory mechanisms of BEOV on neuropathology of the APPSwe/PS1dE9 mice through PPARγ and PTP1B pathways. Further investigation on other pathways is required. Second, we found in this study that BEOV could promote autophagolysosomal fusion and restored autophagic flux. However, its molecular mechanism needs to be investigated. Third, BEOV was found to reduce tau hyperphosphorylation through activation of the IR/IRS-1/PI3K/Akt pathway and inhibition of GSK3β activity in APPSwe/PS1dE9 mice. Other types of AD model mice, such as triple transgenic model mouse of AD (3xTg-AD), should also be used for further evaluating the effect of BEOV on tau hyperphosphorylation.

## Conclusion

BEOV treatment for 3 months at doses of 0.2 and 1.0 mmol/L blocked the amyloidogenic cascade and Aβ deposition in APPSwe/PS1dE9 mice through PPARγ activation. BEOV also inhibited PTP1B expression to increase IR/IRS-1/PI3K/Akt signaling and to inhibit GSK3β activity, thus reducing tau hyperphosphorylation. In addition, BEOV promoted autophagolysosomal fusion and restored autophagic flux to increase the degradation and clearance of Aβ deposits and tau aggregates. Those results provided proof-of-concept evidences of BEOV as a potential novel therapy for the treatment of AD.

## Data Availability Statement

All datasets generated for this study are included in the article/Supplementary Material.

## Ethics Statement

Animal experiments were performed in compliance with the institutional guidelines at Shenzhen University. The protocol was approved by the Laboratory Animal Ethics Committee of Shenzhen University.

## Author Contributions

QL, ZH, NL, XD, and JN designed the study. ZH, HZ, XL, CH, and CW performed the majority of behavioral experiments and analyzed the data. ZH, SH, XH, and QX performed dynamic and static ^18^F-FDG-PET scanning experiments. ZH and HZ performed the *in vitro* experiments and analyzed the data. QL and ZH co-wrote the manuscript. All authors reviewed and concurred with the final manuscript.

## Conflict of Interest

The authors declare that the research was conducted in the absence of any commercial or financial relationships that could be construed as a potential conflict of interest.

## Supplementary Material

FIGURE S1 The effect of BEOV on body weight, liver and renal function of the transgenic AD model mouse APPSwe/PS1dE9. **(A)** The body weights of WT, AD and BEOV-treated AD mice were measured, respectively. (*n* = 12, six females and six males for each group). **(B–D)** Serum was collected from each AD or BEOV-treated AD mouse. The levels of aspartate transaminase (AST), creatinine and urea were measured, respectively. *n* = 6, three females and three males for each group.

FIGURE S2 The levels of vanadium increased both in the serum and brain of APPSwe/PS1dE9 AD mice after 3-month-treatment with BEOV. **(A)** Vanadium levels (ng/mL) in serum were measured after treating with BEOV for 3 months (*n* = 10, five females and five males for each group). **(B)** Vanadium levels (ng/g) in the brain were measured after treating with BEOV for 3 months. *n* = 6, three females and three males for each group. ****P* < 0.001, AD+BEOV group vs. AD group.

FIGURE S3 BEOV inhibited JNK activation in the transgenic AD model mouse APPSwe/PS1dE9. **(A,C)** Western blotting analyses of the p-JNK and JNK levels in the hippocampus and cortex of WT, AD and BEOV-treated AD mice. **(B,D)** Semi-quantification of the relative ratio of p-JNK/JNK in mouse hippocampus and cortex. Quantitative results were normalized against the levels of GAPDH. *n* = 6, three females and three males. ^#^*P* < 0.05, WT group vs. AD group; **P* < 0.05 and ***P* < 0.01, respectively, AD + BEOV group vs. AD group.

TABLE S1 Primary antibody information.

VIDEO S1 ^18^F-FDG uptake in the brain of WT mouse.

VIDEO S2 ^18^F-FDG uptake in the brain of AD mouse.

VIDEO S3 ^18^F-FDG uptake in the brain of AD mouse treated with 0.2 mmol/L BEOV.

VIDEO S4 ^18^F-FDG uptake in the brain of AD mouse treated with 1.0 mmol/L BEOV.
