# Supplementary material for: The Protective Effect of Vanadium on Cognitive Impairment and the Neuropathology of Alzheimer’s Disease in APPSwe/PS1dE9 Mice
Source: Front Mol Neurosci. 2020 Mar 10;13:21. doi: 10.3389/fnmol.2020.00021 (PMC7077345; doi:10.3389/fnmol.2020.00021)
Supplement: FIGURE S1 — The effect of BEOV on body weight, liver and renal function of the transgenic AD model mouse APPSwe/PS1dE9. (A) The body weights of WT, AD and BEOV-treated AD mice were measured, respectively. (n = 12, six females and six males for each group). (B–D) Serum was collected from each AD or BEOV-treated AD mouse. The levels of aspartate transaminase (AST), creatinine and urea were measured, respectively. n = 6, three females and three males for each group. [file Data_Sheet_1.docx]

**Supplementary Data**

**Supplementary Table**

**Table 1**

Primary antibody information

| **Antibody** | **Host** | **Application** | **Source** | **Identifier** |
| --- | --- | --- | --- | --- |
| PSD95 | Rabbit | WB (1:5000) | Abcam | Cat#ab18258 |
| Synaptophysin | Rabbit | WB (1:10000) | Abcam | Cat#ab32127 |
| APP | Rabbit | WB (1:10000) | Abcam | Cat#ab32136 |
| BACE1 | Rabbit | WB (1:3000) | Abcam | Cat#ab108394 |
| sAPPβ | Rabbit | WB (1:500) | Biolegend | Cat#813401 |
| Aβ | Mouse | WB (1:500) | Biolegend | Cat#803002 |
| IDE | Rabbit | WB (1:3000) / IF (1:100) | Abcam | Cat#ab109538 |
| PPARγ | Rabbit | WB (1:1000) | Cell Signaling | Cat#2443 |
| HT5 | Mouse | WB (1:1000) | Abcam | Cat#ab80579 |
| Tau-pThr231 | Rabbit | WB (1:5000) | Abcam | Cat#ab151559 |
| Tau-pSer396 | Rabbit | WB (1:10000) | Abcam | Cat#ab109390 |
| Tau-pSer422 | Rabbit | WB (1:5000) | Abcam | Cat#ab79415 |
| Tau-pSer404 | Rabbit | WB (1:2000) | Abcam | Cat#ab92676 |
| PTP1B | Rabbit | WB (1:1000) | Cell Signaling | Cat#5311 |
| AKT | Rabbit | WB (1:1000) | Abcam | Cat#ab8805 |
| p-AKT | Rabbit | WB (1:1000) | Abcam | Cat#ab81283 |
| GSK3β | Rabbit | WB (1:5000) | Abcam | Cat#ab32391 |
| GSK3β pY216 | Rabbit | WB (1:3000) | Abcam | Cat#ab75745 |
| GSK3β pSer9 | Rabbit | WB (1:5000) | Abcam | Cat#ab131079 |
| GAPDH | Rabbit | WB (1:5000) | Proteintech | Cat#10494-1-AP |
| LC3 | Rabbit | WB (1:1000) | Cell Signaling | Cat#12741 |
| CathepsinD | Rabbit | WB (1:1000) / IF (1:200) | Cell Signaling | Cat#2284 |
| SQSTM1/p62 | Rabbit | WB (1:1000) | Cell Signaling | Cat#5114 |
| JNK | Rabbit | WB (1:1000) | Cell Signaling | Cat#9252 |
| p-JNK | Rabbit | WB (1:1000) | Cell Signaling | Cat#4668 |
| IR | Rabbit | WB (1:1000) | Cell Signaling | Cat#3025 |
| p-IR | Rabbit | WB (1:1000) | Cell Signaling | Cat#2969 |
| PI3K | Mouse | WB (1:1000) | Abcam | Cat#ab86714 |
| p-PI3K | Rabbit | WB (1:1000) | Cell Signaling | Cat#4228 |
| IR-S1 | Rabbit | WB (1:1000) | Cell Signaling | Cat#2382 |
| p-IR-S1 | Rabbit | WB (1:1000) | Cell Signaling | Cat#2386 |

**Supplementary Figures**

**Supplementary Fig. 1**

**
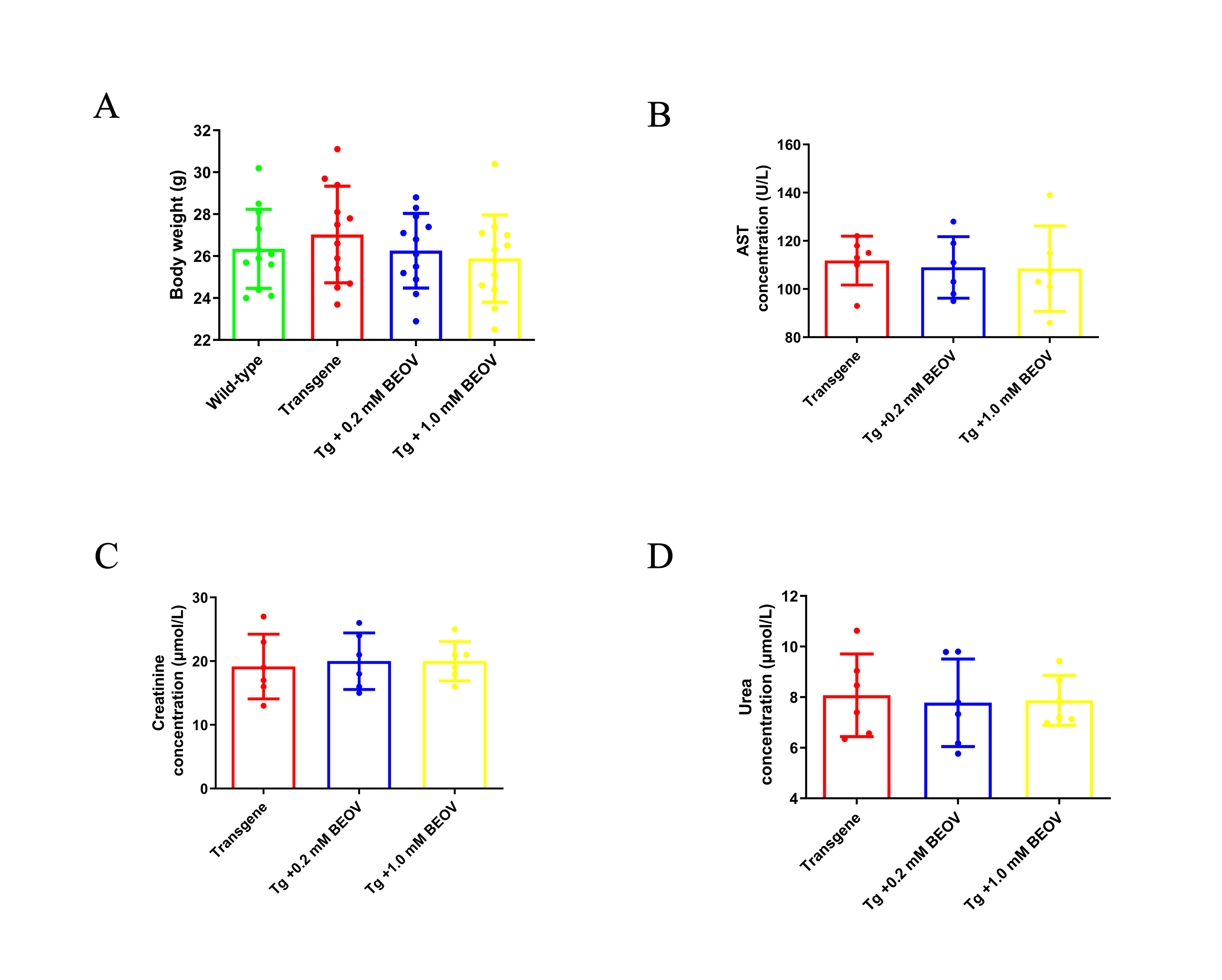
**

**Supplementary Fig. 1 The effect of BEOV on** **body weight,** **liver and renal function of the transgenic AD model mouse APPSwe/PS1dE9.** **(A)** The body weights of WT, AD and BEOV-treated AD mice were measured, respectively. (n=12, six females and six males for each group). **(B-D)** Serum was collected from each AD or BEOV-treated AD mouse. The levels of aspartate transaminase (AST), creatinine and urea were measured, respectively. n=6, three females and three males for each group.

**Supplementary Fig. 2**


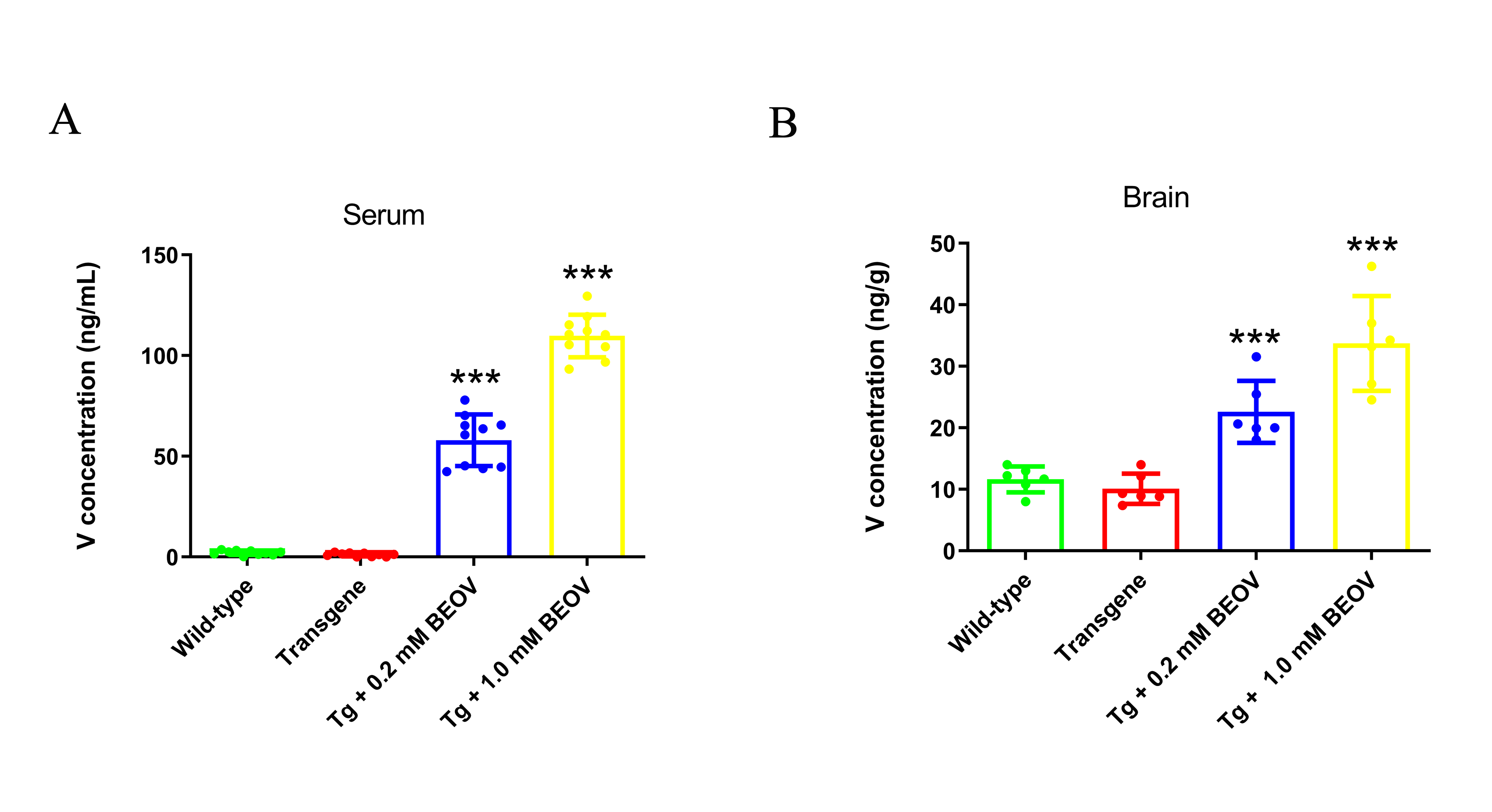


**Supplementary Fig. 2 The levels of vanadium increased both in the serum and brain of** **APPSwe/PS1dE9 AD mice after 3-month-treatment with BEOV.**

**(A)** Vanadium levels (ng/mL) in serum were measured after treating with BEOV for 3 months (n=10, five females and five males for each group). **(B)** Vanadium levels (ng/g) in the brain were measured after treating with BEOV for 3 months. n=6, three females and three males for each group. ****P*<0.001, AD+BEOV group vs AD group.

**Supplementary Fig. 3**

**
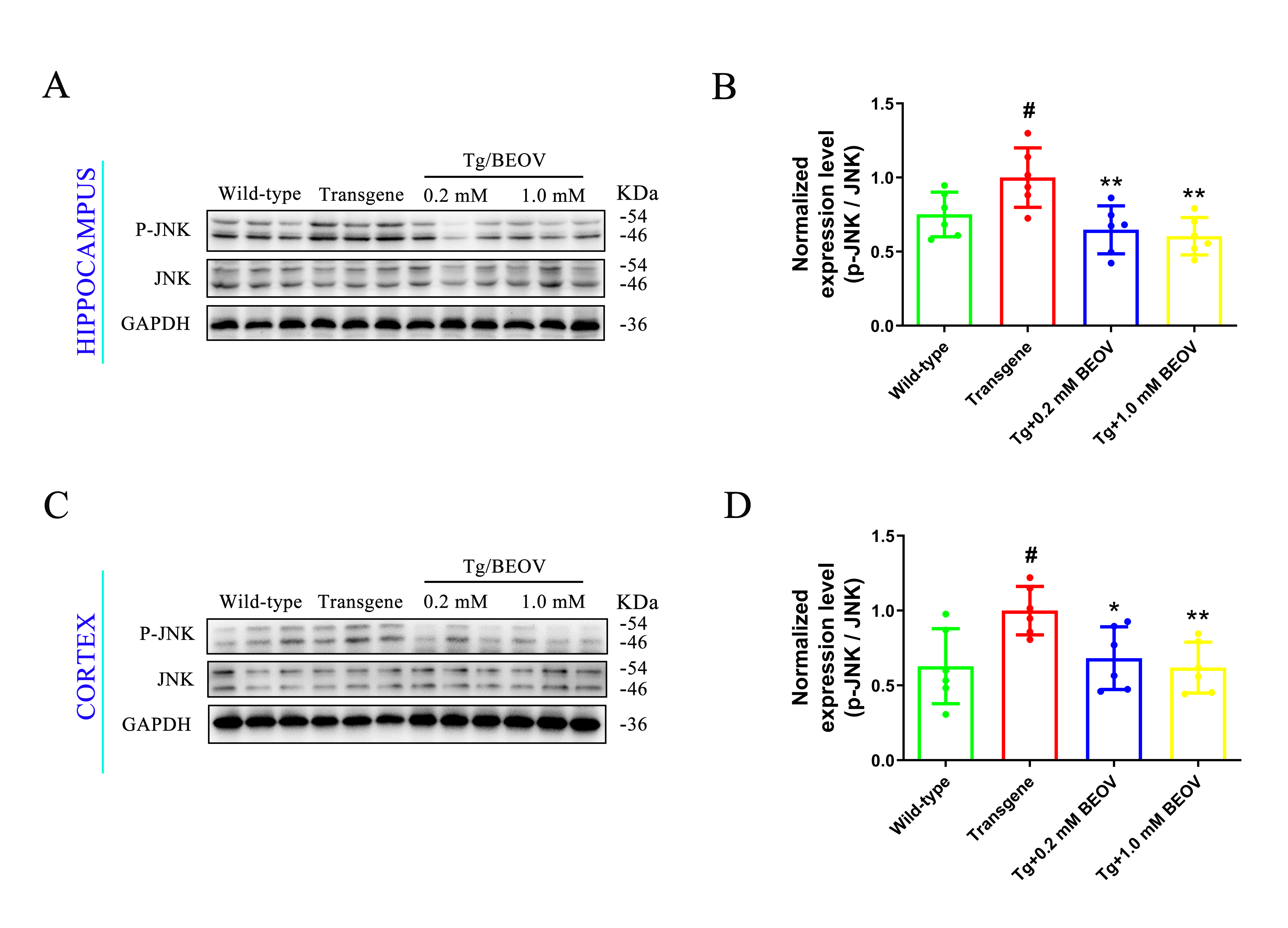
**

**Supplementary Fig. 3. BEOV inhibited JNK activation in the transgenic AD model mouse APPSwe/PS1dE9. (A, C)** Western blotting analyses of the p-JNK and JNK levels in the hippocampus and cortex of WT, AD and BEOV-treated AD mice. (**B, D)** Semi-quantification of the relative ratio of p-JNK/JNK in mouse hippocampus and cortex. Quantitative results were normalized against the levels of GAPDH. n=6, three females and three males. ^#^*P*<0.05, WT group vs AD group; **P*<0.05 and ***P*<0.01 respectively, AD+BEOV group vs AD group.
